# Editorial: Advances in explainable analysis methods for cognitive and computational neuroscience

**DOI:** 10.3389/fnins.2026.1938732

**Published:** 2026-08-18

**Authors:** Guoyang Liu, Yueyuan Zheng, Jinxiao Zhang, Lan Tian, Weidong Zhou

**Affiliations:** 1 Shenzhen Research Institute of Shandong University, Shenzhen, China; 2 School of Integrated Circuits, Shandong University, Jinan, China; 3 Division of Social Science, The Hong Kong University of Science and Technology, Hong Kong, Hong Kong SAR, China; 4 Department of Neurology, University of California, San Francisco, San Francisco, CA, United States

**Keywords:** biomarkers, brain-computer interfaces, cognitive neuroscience, computational neuroscience, EEG decoding, explainable artificial intelligence, interpretability, neurotechnology

Artificial intelligence (AI) is being increasingly integrated into cognitive and computational neuroscience to support the preprocessing, analysis, and interpretation of multimodal data, including electroencephalography (EEG), neuroimaging, speech, wearable-sensor, behavioral, and molecular data. Today, high predictive accuracy alone is insufficient when models are used to infer neural mechanisms, stratify patients, or develop neurotechnologies. Therefore, explainable analysis methods play an important role in understanding the neural signals used by an AI model and converting statistical regularities into hypotheses about physiology, cognition, and intervention targets. Contemporary explainable AI (XAI) spans both *ante-hoc* (e.g., sparse, rule-based, mechanistic, or geometry-aware models) and *post-hoc* (e.g., feature importance, saliency maps, layer-wise relevance propagation, Grad-CAM, SHAP, and counterfactual analysis) approaches (Bach et al., 2015; Ribeiro et al., 2016; Lundberg and Lee, 2017; Selvaraju et al., 2017; Barredo Arrieta et al., 2020). In neuroscience, these tools must also be tested for biological plausibility, spatiotemporal stability, sensitivity to preprocessing, interindividual variability, and out-of-sample generalization. For example, EEG decoding and visualization have shown how learned features can be mapped back to oscillatory and topographic patterns (Schirrmeister et al., 2017), and recent EEG decoding research in face recognition has linked SVM-based neural representational dynamics with interpretable eye movement patterns and consistency (Liu et al., 2025). This Research Topic was organized precisely around this need: to move from black-box prediction toward transparent, reproducible, and mechanistically informative analyses in cognitive and computational neuroscience.

The nine contributions collected here demonstrate that explainability is not a single technique but rather a multi-methodological stance that connects modeling choices to neuroscientific meaning. In the domain of EEG analysis, Lai et al. used covariance-based Riemannian decoding algorithms to differentiate sleep spindle responses to declarative and non-declarative odor cueing, providing explainable channel-level contribution maps. Extending this explainable modeling logic to motor imagery brain-computer interfaces (BCIs), Zheng et al. combined wavelet-packet synthetic augmentation, entropy-based channel selection, and a lightweight, multi-branch network to improve decoding performance while reducing sensor burden. Their study illustrates how interpretable channel selection can support deployable BCI design. Complementing these task-specific neural decoding methods, Huang et al. analyzed phase-amplitude coupling (PAC) in EEG data during auditory oddball processing in disorders of consciousness and found delta-gamma PAC predominance with an emphasis that interpretable oscillatory markers are paradigm-dependent rather than universal signatures. Two speech-related studies extended explainable AI methods beyond conventional neural data: Cheng et al. used Grad-CAM and SHAP to demonstrate that a CNN-LSTM forensic speech model focuses on high-frequency artifacts and temporal inconsistencies, whereas Shi et al. embedded automatic speech recognition and speaker verification within a knowledge-driven checklist framework for surgical safety, showing how domain constraints can make AI decisions more auditable in noisy real-world settings.

A second group of contributions highlights the translational value of explainable AI when it is paired with domain knowledge and validation. In the domain of clinical predictive modeling, Li et al. integrated wearable IMU and sEMG signals with musculoskeletal kinetic modeling to derive phase-specific biomarkers for post-stroke upper-limb dysfunction; Lasso prediction of FMA-UL scores was interpreted with SHAP, identifying trunk displacement and inter-joint coordination as clinically meaningful targets for precision rehabilitation. Liu et al. combined bioinformatics, random forest, SVM-RFE, LASSO, molecular docking, and animal validation to identify TLR2 and CYP1B1 as inflammation-related hub genes in post-stroke depression and to support the therapeutic relevance of berberine. Wu et al. reviewed the role of microglial TREM2 as a bridge between Alzheimer's disease, spinal cord injury-related cognitive impairment, and AI-assisted therapeutic discovery. The authors underscored how explainable, multimodal models can connect molecular states, neuroimaging, and clinical trajectories. Beyond clinical and biomedical contexts, the explainable AI framework can also be extended to behavioral and cognitive dynamic tracking. Li and Zhu proposed a novel Cognitive Drift Index for the auditing of AI-driven recommendation environments, thereby expanding the scope of cognitive neuroscience from internal neural computation to the algorithmically curated information ecologies that shape attention, beliefs, and health-related behaviors.

Taken together, these studies highlight several priorities for the next stage of explainable AI in neuroscience. First, explanations should be evaluated for robustness: attribution maps, selected channels, and biomarker rankings should be tested for robustness across folds, preprocessing pipelines, cohorts, hardware, and perturbations. Second, explanations should be multilevel, linking features to interpretability at the scale of brain regions, time-frequency structure, muscles, genes, behavior, and clinical endpoints. Third, causal inference and intervention are needed to separate correlational relevance from mechanistic importance, especially in clinical and closed-loop neurotechnology settings. Finally, model transparency must be accompanied by uncertainty quantification, data governance, and human-in-the-loop evaluation, because explanations that look plausible can still be unfaithful or biased (Rudin, 2019). The contributions in this Research Topic collectively show that explainable analysis methods can make AI-accelerated neuroscience more rigorous, credible, and productive. Treating interpretability as a design priority, cognitive and computational neuroscience can transform predictive models into neurotechnology for discovery, diagnosis, and intervention.
